# Robertsonian chromosomes and the nuclear architecture of mouse meiotic prophase spermatocytes

**DOI:** 10.1186/0717-6287-47-16

**Published:** 2014-05-14

**Authors:** Soledad Berríos, Catalina Manieu, Julio López-Fenner, Eliana Ayarza, Jesús Page, Marisel González, Marcia Manterola, Raúl Fernández-Donoso

**Affiliations:** Programa Genética Humana, ICBM, Facultad de Medicina, Universidad de Chile, Santiago, Chile; Departamento de Ingeniería Matemática, Universidad de La Frontera, Temuco, Chile; Departamento de Biología Celular, Universidad Autónoma de Madrid, Madrid, España

**Keywords:** Robertsonian chromosomes, Mouse spermatocytes, Bivalent associations, Nuclear architecture

## Abstract

**Background:**

The nuclear architecture of meiotic prophase spermatocytes is based on higher-order patterns of spatial associations among chromosomal domains from different bivalents. The meiotic nuclear architecture depends on the chromosome characteristics and consequently is prone to modification by chromosomal rearrangements. In this work, we consider *Mus domesticus* spermatocytes with diploid chromosome number 2n = 40, all telocentric, and investigate a possible modification of the ancestral nuclear architecture due to the emergence of derived Rb chromosomes, which may be present in the homozygous or heterozygous condition.

**Results:**

In the 2n = 40 spermatocyte nuclei random associations mediated by pericentromeric heterochromatin among the 19 telocentric bivalents ocurr at the nuclear periphery. The observed frequency of associations among them, made distinguishable by specific probes and FISH, seems to be the same for pairs that may or may not form Rb chromosomes. In the homozygote Rb 2n = 24 spermatocytes, associations also mediated by pericentromeric heterochromatin occur mainly between the three telocentric or the eight metacentric bivalents themselves. In heterozygote Rb 2n = 32 spermatocytes all heterochromatin is localized at the nuclear periphery, yet associations are mainly observed among the three telocentric bivalents and between the asynaptic axes of the trivalents.

**Conclusions:**

The Rb chromosomes pose sharp restrictions for interactions in the 2n = 24 and 2n = 32 spermatocytes, as compared to the ample possibilities for interactions between bivalents in the 2n = 40 spermatocytes. Undoubtedly the emergence of Rb chromosomes changes the ancestral nuclear architecture of 2n = 40 spermatocytes since they establish new types of interactions among chromosomal domains, particularly through centromeric and heterochromatic regions at the nuclear periphery among telocentric and at the nuclear center among Rb metacentric ones.

## Background

During the meiotic prophase of spermatocytes, chromosomes form a distinctive arrangement in the nuclear space, governed mainly by the synapses between homologous chromosomes that form bivalents and by the union of their ends to the nuclear envelope [1–3]. The bivalent configuration generates a meiotic nuclear architecture dependent on the chromosome characteristics [4–6]. Consequently, this architecture is subject to modification by chromosomal rearrangements.

In this work, we investigate how the ancestral nuclear architecture of *Mus domesticus* spermatocytes with a diploid chromosome number 2n = 40, all of them telocentric, is modified by the emergence of derived Rb metacentric chromosomes, which may be present in the homozygous or heterozygous condition.

Synapsis between homologous chromosomes is a process that culminates with the formation of the synaptonemal complex (SC). The SC is a tripartite proteinaceous scaffold consisting of two lateral elements (homologous chromosome axes) and a medial component that stabilizes the joint between the homologous chromosomes forming the bivalents [7–10].

Numerous proteins are involved in the SC structure. SYCP3 protein is the main constituent of the axis of each homologous chromosome, and SYCP1 protein is located between the axes of homologous chromosomes, binding them together in synapsis with transverse filaments [11, 12]. Both ends of each bivalent SC are attached to the nuclear envelope, so that each SC forms an arc of different extension depending on the length of the synapsed chromosomes. The chromatin, organized in loops, is connected to the lateral elements of the SC, hence the chromatin domains are sequentially ordered along each bivalent SC. Finally, the SC’trajectory determines the place for the chromosomal domains to occupy within the nuclear space. This is not trivial, because interactions or associations among heterologous chromosomal domains depend on the real possibility of establishing contacts between them together with their structural and functional affinities that could favor the consolidation of such interactions.

In this regard, the spermatocytes of *Mus domesticus* 2n = 40 containing 19 autosomal telocentric bivalents with abundant pericentromeric heterochromatin near their proximal ends naturally favor the associations among them over the nuclear envelope [4]. Comparative analysis of the observed combinations of associated and non-associated bivalents in the spermatocytes, and the predictions of an ad-hoc developed probabilistic model for associations between indistinguishable elements, suggest that these associations could indeed take place randomly [6]. Notice that associations are not a phenomenon unique to meiotic prophase cells. They have also been described in *Mus* somatic cells with highly complex chromocenters, involving several chromosomes and the nucleoli [13].

Large blocks of satellite DNA (pericentromeric heterochromatin) surround the centromere and extend towards the proximal end of each chromosome, thus favoring the occurrence of Rb translocations in *Mus*. Double-strand breaks of DNA with nearly identical base components, in addition to physical proximity, facilitates the fusion of different chromosomes [14]. Rb translocations involve double-strand DNA breaking at the centromere level in two telocentric (acrocentric) chromosomes, followed by a repair (fusion) that binds the respective long arms, creating a metacentric Rb chromosome. The short arms (p) of the original telocentric chromosomes, including the proximal telomeres, part of the satellite DNA, and generally one centromere, are all lost. However, this loss of DNA does not significantly alter the total amount of DNA as compared with the standard *Mus* karyotype [15]. Rb translocation is the most common chromosomal rearrangement in mammals [16] and represents the type of chromosomal change that most effectively contributes to differentiation or speciation of natural populations [17].

In *Mus*, Rb translocations have resulted in more than 40 different chromosomal races (or subspecies), ranging from 2n = 40 to 2n = 22. These chromosomal races are natural populations characterized by altogether about 100 Rbs chromosomes with different combinations of arms [18], many of which emerged and spread extremely rapidly within populations of the standard karyotype [19].

Metacentric Rb chromosomes can become numerous in the *Mus* genome, leading to a reduction of ancestral telocentric chromosomes and to an emergence of new mixed karyotypes. Furthermore, crossing between wild homozygotes 2n = 40 and Rb homozygotes produce F1 hybrids in whose genomes the ancestral telocentric chromosomes are reunited with the metacentric derivatives. Trivalents are formed in the meiotic prophase spermatocytes of these mice, in which a metacentric chromosome is synapsed with the long arms of two telocentric chromosomes. Varying degrees of synapses are established among the proximal ends of the involved telocentric chromosomes, despite being heterologous [20].

The presence of Robertsonian (Rb) metacentric chromosomes, which can be multiple in this species, constitutes a valuable opportunity to study how these new chromosomes modify the original, ancestral nuclear architecture built just for telocentric bivalents.

To this end, we studied the meiotic nuclear organization of:

- Homozygote 2n = 24 spermatocytes, with 8 pairs of metacentric Rb chromosomes, 3 pairs of telocentric chromosomes, and the XY sex pair.
- Heterozygote 2n = 32 spermatocytes, with 8 Rb metacentric chromosomes, 22 telocentric chromosomes, and the XY pair.

We further compared both families of spermatocytes with the nuclear architecture of the ancestral homozygote 2n = 40.

We found different patterns of nuclear architecture according to chromosome constitution of the spermatocytes. The Rb chromosomes present in 2n = 24 and 2n = 32 spermatocytes drastically restrict the possibilities for interaction between the heterochromatic domains as compared with the wealth of random associations observed in 2n = 40 spermatocytes.

## Results

### Early prophase and the chromosome nuclear distribution in 2n = 40 and 2n = 24 spermatocytes

**Figure 1:**
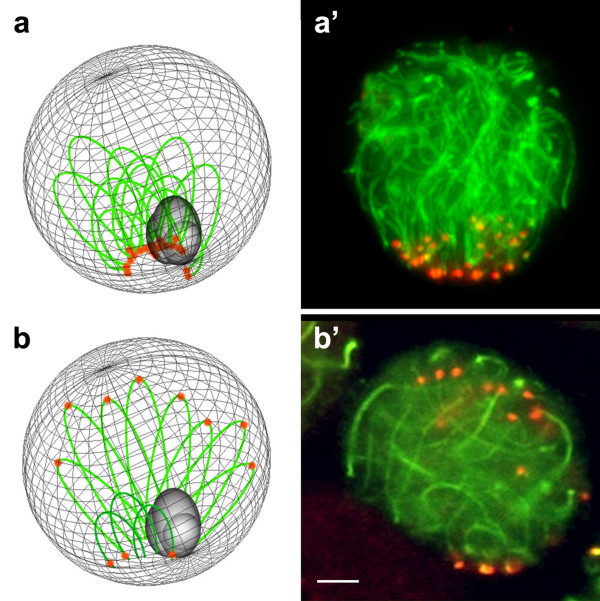
**Bouquet configuration in early meiotic prophase nuclei of 2n = 40 and 2n = 24 spermatocytes.** Diagram **(a)** and nucleus **(a')** showing the configuration of autosomal chromosomes in the “bouquet” of a 2n = 40 spermatocytes. The chromatin of the sex bivalent is also represented (a). The centromeres (red) aggregate in one main group at the nuclear periphery, while the chromosomal axes (green) describe arches toward the nuclear space. Diagram **(b)** and nucleus **(b')** showing the configuration of autosomal chromosomes in the “bouquet” of a 2n = 24 spermatocyte. The chromatin of the sex bivalent is also represented (b). The centromeres appear in two major clusters: one at the nuclear periphery, grouping the centromeres of the telocentric bivalents, and the other toward the nucleus center, grouping the centromeres of the metacentric Rb bivalents. The axes that emerge from the peripheral centromeres are shorter than those linked to the central centromeres. The chromosomal axes (green) and centromeres (red) in nuclei with preserved sphericity from 2n = 40 and 2n = 24 spermatocytes were identified by immunochemistry using, respectively, anti-SYCP3 and anti-CENPA antibodies. Bar = 5 μm.

In early prophase nuclei, chromosome/bivalents are aggregated in a configuration known as the bouquet, in which the telomeric regions of all chromosomes cluster over a small area of the nuclear envelope. In this meiotic stage, the 2n = 40 spermatocytes showed centromeres aggregated in one or two main groups at the nuclear periphery, while the respective axes or SCs described arcs toward the nuclear space (Figure 1a & a’). In the bouquet configuration of the 2n = 24 spermatocytes, the centromeres appeared in two major clusters: one at the nuclear periphery, grouping the centromeres of the telocentric bivalents, and the other one toward the nucleus center, grouping the centromeres of the metacentric Rb bivalents (Figure 1b & b’).

When the early prophase nuclei were treated with DAPI that stains DNA rich in AT sequences, which in *Mus* is the DNA that underlies the pericentromeric heterochromatin, the 2n = 40 spermatocyte nucleus showed one large DAPI-positive chromocenter whose location matched the regions of aggregated centromere. The 2n = 24 spermatocytes showed at least two DAPI-positive chromocenters, one at the nuclear periphery and the other toward the center of the nucleus. We showed previously that the initial aggregation of centromeres occurring in lepto-zygotene of 2n = 40 spermatocytes will eventually resolve into several smaller aggregates that we can observe in pachytene or more advanced stages of meiotic prophase [6].

### Bivalent configuration and associations in 2n = 40 pachytene spermatocytes

**Figure 2:**
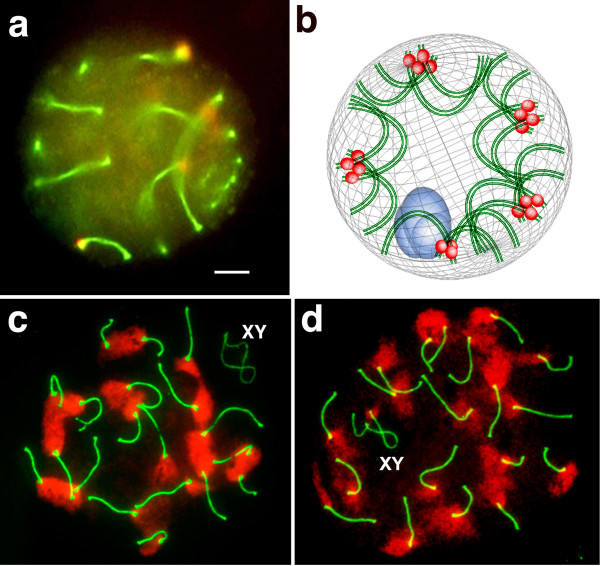
**Nuclear architecture of spermatocytes 2n=40.** **a** Distribution of telocentric bivalents in a 3D pachytene nucleus from 2n = 40 spermatocytes. The synaptonemal complexes (green) and centromeres (red) were identified by immunochemistry using anti-SYCP3 and anti-CENPA antibodies. The 19 autosomal bivalents form regular arcs with the centromeres (red) (and the heterochromatin) in one extreme of each and consequently localized at the nuclear periphery. The focus is over one telocentric bivalent. Bar = 5 μm. **b** Scheme representing the nuclear architecture of 2n = 40 spermatocytes. The centromeric regions (red) of all telocentric bivalents are located at the nuclear periphery. The bivalentes can be alone or associated in different numbers. The XY chromatin is also represented (blue). **c & d**. Associations between telocentric bivalents through heterochromatin in 2n = 40 spermatocytes. The SC (green) and the pericentromeric heterochromatin (red) were identified by immunochemistry using, respectively, anti-SYCP3 and anti-H3K9me3 antibodies. XY = sex bivalent. The19 autosomal bivalents appear singly or associated in different numerical combinations per spermatocyte. The combinatory presents in the nucleus of figure c) is: 5-4-3-2-2-2-1 = 19; and in the figure d) is: 6-3-2-2-2-1-1-1-1 = 19.

**Figure 3:**
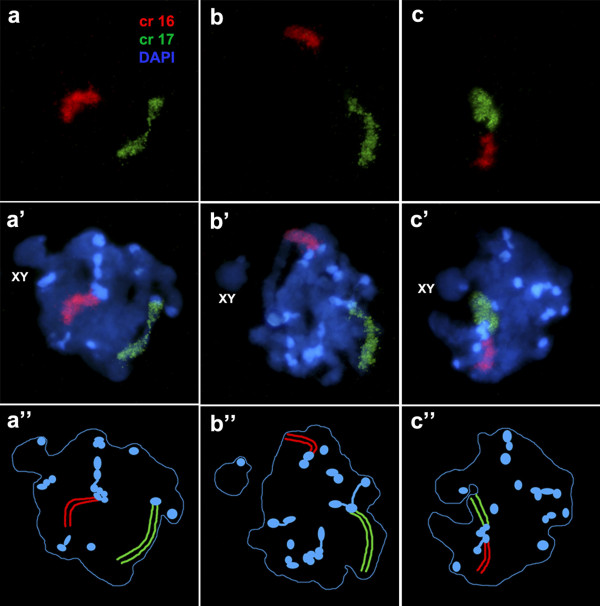
**Associations between specific bivalents in pachytene nuclear squashs from 2n = 40 spermatocytes. a, b, and c**: The bivalents 16 (red) and 17 (green) appear identified by FISH and specific probes. **a’, b’ and c’**: Same nuclei counterstained with DAPI; **a”, b” and c”**: Representation of the painted bivalents 16 and 17 and the DAPI heterochromatin of the all bivalents. In the nucleus a”, the bivalent 16 through the heterochromatin is associated with 5 bivalents while the bivalent 17 is single; In the nucleus b”, the bivalent 16 as the bivalent 17 are independently associated with other 2 bivalents; in the nucleus c”, both bivalents 16 and 17 are associated with each other and with two more bivalents through their heterochromatins.

In these meiotic nuclei all the telocentric SCs described regular arcs with the centromeres located in one extreme near the nuclear envelope. Figure 2a shows a well-preserved pachytene nucleus with this chromosomal configuration. The focal plane is over the SC of one of the smallest bivalent where the centromeric domain close to the nuclear limit can be distinguished (Figure 2a). The abundant pericentromeric heterochromatin in all the bivalents preserving associations among them, produce chromocenters of different sizes, scattered throughout the nuclear envelope (Figure 1b). Therefore, each chromocenter is corresponds to a cluster of pericentromeric heterochromatin formed by the joint contribution of all those bivalents that remain associated between them throughout the prophase. The size of the chromocenter was proportional to the number of the associated bivalents. In pachytene nuclear spreads the clusters of heterochromatin were well preserved. By immunochemistry, both the axial elements of the SC as well as the pericentromeric heterochromatin, could be identified and, consequently, the number of bivalents participating in each chromocenter could be determined (Figure 2c & d). The associations among chromosomes were studied in 100 microspreads of 2n = 40 pachytene spermatocytes, considering the 19 autosomal bivalents as indistinguishable elements from one another. Each nucleus exhibited different combinations of chromosomal clusters. More than 60% of the pachytene spermatocytes presented groups of 4, 5 or 6 bivalents, as the highest number of associated bivalents (Figure 2c & d). Spermatocytes with no associations among bivalents or forming a cluster involving more than 11 associated bivalents were never observed (See [6]). Besides, we also studied the associative behavior of specific bivalents, identifying by FISH and chromosomal probes two bivalents at a time in 100 spermatocytes each (Figure 3). The probes were specific for chromosomes 9, 14, 16, and 17, and they were chosen because the chromosome pairs 9/14 and 16/17 form Rb metacentric chromosomes. In approximately 60% of spermatocytes, the two marked bivalents were found to be associated with other bivalents in a number of combinations, but not among themselves (Figure 3a & b). In approximately 30 to 32% of the spermatocytes, the identified bivalents were alone, not associated with each other or with other bivalents. In about 8% to 11% of the studied spermatocytes, the two identified bivalents were associated to each other (Figure 3c). No differences was observed in the frequency of association between pairs of chromosomes that may form Rb metacentric chromosomes (9/14, 16/17), as compared to those pairs that do not form Rb chromosomes (9/17, 14/16) were observed (See Table 1). A computer simulation of all possible associations between the 19 elements, which included only 2 distinguishable bivalents, revealed that the frequency of events in which they can be associated with each other over all conditions was close to 12% (López-Fenner J, Berrios S, Manieu C, Page J, Fernández-Donoso R. Bivalent associations in Mus domesticus 2n = 40 spermatocytes. Are they random? Submitted). If we use this theoretical value as a reference, we can consider that the observed frequencies of association between distinguishable elements could also be random, at least for the four pairs of bivalents here studied.

**Table 1:** **Frequency of 2n = 40 spermatocyte bearers with two given distinguished bivalents associated between them**

Pair of distinguishable bivalents	Spermatocyte percentage with those bivalents associated between them
16 and 17	8
9 and 14	10
14 and 16	11
9 and 17	10

One hundred spermatocyte nuclei for each pair of bivalents were scored.

The nuclei were stained with DAPI, and the specific bivalents were identified by FISH and specific DNA probes.

### Bivalent configuration and associations in 2n = 24 pachytene spermatocytes

**Figure 4:**
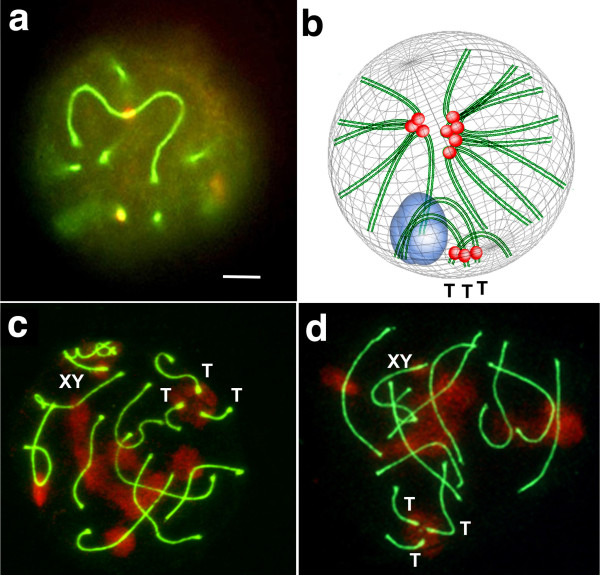
**Nuclear architecture of spermatocytes 2n=24.** **a** Distribution of metacentric and telocentric bivalents in a 3D pachytene nucleus from 2n = 24 spermatocytes. The synaptonemal complexes (green) and centromeres (red) were identified by immunochemistry using anti-SYCP3 and anti-CENPA antibodies. The metacentric bivalents form SCs longer than those of the telocentric bivalents and the centromeres are localized in the middle of the metacentric bivalents unlike the terminal position in the telocentric bivalents. The focus is over one metacentric Rb bivalent. Bar = 5 μm. **b**. Scheme representing the nuclear architecture of 2n = 24 spermatocytes. The centromeric regions of the three telocentric bivalents (T) are located at the nuclear periphery and the centromeric regions of the metacentric bivalents at the nuclear center. The associations are produced mostly within groups of telocentric or metacentric bivalents. The XY chromatin is also respresented (blue). **c** &**d** Associations between metacentric and telocentric bivalents through heterochromatin in 2n = 24 spermatocytes. The SC (green) and the pericentromeric heterochromatin (red) were identified by immunochemistry using anti-SYCP3 and anti-H3K9me3 antibodies. The associations were mainly between the telocentric or the metacentric bivalents. c) Nucleus with 6 associated metacentric bivalents and 2 singles; the 3 telocentric bivalents appear all together. The XY bivalent is indicated. d) Nucleus with three groups of metacentric bivalents: 4 associated, 2 associated and 1 single; the 3 telocentric bivalents appear all together. The XY bivalent is indicated.

In these nuclei only three SCs described regular arcs with the centromeres localized in one extreme near the nuclear envelope because only three autosomal bivalents are even telocentrics. The eight metacentric Rb bivalents formed longer SCs than those of the telocentric bivalents and the centromeres were localized in the middle of the metacentric Rb bivalents, far from the nuclear envelope. In a well-preserved nucleus the focus was over one metacentric Rb bivalent (Figure 4a). In 2n = 24 spermatocytes, associations were also produced through the pericentromeric heterochromatin, but mostly within groups of telocentric or metacentric bivalents (Figure 4b). Nuclear spreads with well-preserved clusters of heterochromatin showed two or more groups of metacentric bivalents associated in different number combinations and the telocentric bivalents mainly associated among themselves (Figure 4c & d). The nuclear frequency per each chromosomal combination is shown in Table 2. In 27% of the spermatocytes the 3 telocentric bivalents were all together, in 56% of the nuclei only two bivalents were found associated and one single; and in approximately 17% of the nuclei the 3 telocentric bivalents were not in association, neither among themselves nor with other bivalents. Only 12% of the examined spermatocytes also showed associations between telocentric and metacentric bivalents (Table 2). In about 19% of the spermatocytes some bivalents were associated with the XY bivalent.

**Table 2:** **Combination of associated and single metacentric/telocentric bivalents per nucleus observed in 2n = 24 spermatocytes**

Combinations between 8 metacentric bivalents	Combinations between 3 telocentric bivalents	Metacentric/Telocentric associations	Number of spermatocytes per nuclear combination	% of spermatocytes per Class
8-0	- - -		0	0
7-1	2(3); 5(2-1)		7	7
6-2	3(3); 2(2-1)		5	12
6-1-1	2(3); 5(2-1)	1(1M2T)	7	
5-3	1(1-1-1)	1(3M1T)	1	8
5-2-1	1(3); 3(2-1)		4	
5-1-1-1	2(3);1(1-1-1)		3	
4-4	1(2-1);2(1-1-1)		3	31
4-3-1	3(3); 9(2-1); 1(1-1-1)		13	
4-2-2	2(3); 3(2-1); 1(1-1-1)	1(2M2T)	6	
4-2-1-1	2(3); 4(2-1); 2(1-1-1)	1(1M1T)	8	
4-1-1-1-1	1(1-1-1)		1	
3-3-2	1(3); 4(2-1); 2(1-1-1)	1(1M1T)	7	38
3-3-1-1	3(3); 4(2-1); 3(1-1-1)	1(3M1T)	10	
3-2-2-1	4(3); 8(2-1); 2(1-1-1)	1(1M1T)	14	
3-2-1-1-1	1(3); 4(2-1);	1(1M1T)	5	
3-1-1-1-1-1	2(2-1)	1(3M1T) 1(1M1T)	2	
2-2-2-2				4
2-2-2-1-1	1(3): 2(2-1);1(1-1-1)	1(2M2T);1(1M1T)	4	
2-2-1-1-1-1				
2-2-1-1-1-1				
2-1-1-1-1-1-1				
1-1-1-1-1-1-1-1				0
TOTAL	27(3); 56(2-1); 17(1-1-1)	12	100	100

100 nuclear microspreadings from 2n = 24 spermatocytes were examined.

The SC and the pericentromeric heterochromatin were identified by immunochemistry using, respectively, anti-SYCP3 and anti-H3K9me3 antibodies (as in Figure 4c & d).

The first column shows the combination of associated and single metacentric bivalents per nucleus; The 2^nd^ column the combination of associated and single between 3 telocentric bivalents; the 3th column the spermatocytes that showed associations between metacentric and telocentric bivalents; The 4^th^ column the frequency of spermatocytes per nuclear combination; and the 5^th^ column the frequency of spermatocytes per Class, considering as such, all the nuclei sharing the same highest combination of metacentric bivalent.

For example the third line of this Table means: In 5 spermatocytes the metacentrics were in a group of 6 and in another of two bivalents. In 3 of these spermatocytes the telocentrics were all together and in 2 they were in a combination 2 together and one alone. 12 spermatocytes were Class 6 or 12 had 6 as the higher number of associated metacentric bivalents.

### Trivalent configurations in spermatocyte nuclei of heterozygotes 2n = 32

**Figure 5:**
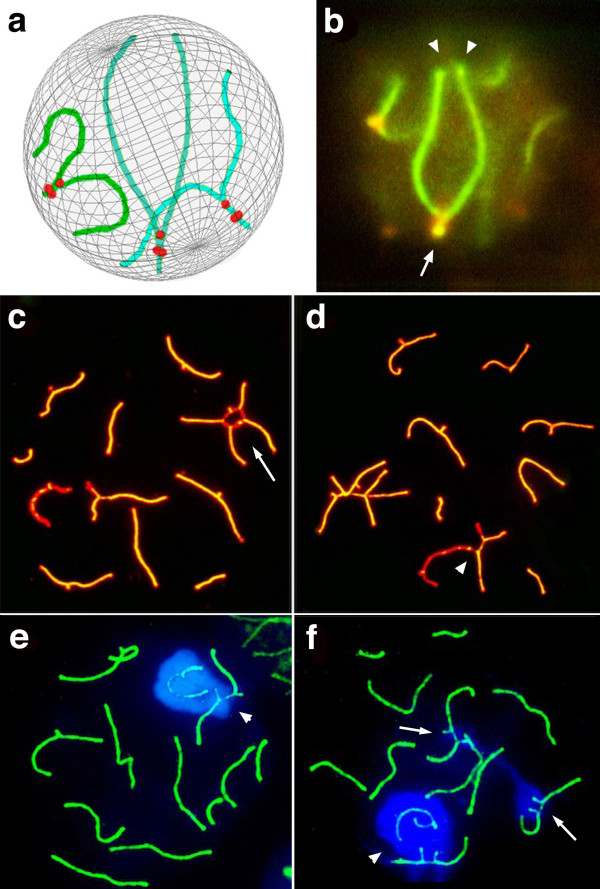
**Configurations of synapsed trivalents in pachytene nuclei of 2n = 32 spermatocytes (a–b) and ectopic associations between asynapsed trivalents and the XY bivalent (d–f). a–b**.Trivalent configurations in a diagram (a) and in a well preserved nucleus (b). The synaptonemal complexes (green) and centromeres (red) were identified by immunochemistry, using, respectively, anti-SYCP3 and anti-CENPA antibodies. The trivalents present 3 points of attachment to the nuclear envelope, two of which correspond to the distal telomeres of the metacentric chromosomes synapsed with the long arms of the telocentric chromosomes (arrowheads), and the third to the attachment of the proximal telomeres of the telocentric chromosomes (arrow). The third point drags the pericentromeric heterochromatin of the 3 chromosomes involved in the trivalent towards the nuclear periphery. **c–d**. In nuclear spreads the chromosomal axes (red) and central element of the SC (green) were identified by immunochemistry, using, respectively, anti-SYCP3 and anti-SYCP1 antibodies. c) Two asynapsed trivalents are associated through both single axes from the two telocentric short arms (arrow). In both bonding zones, the protein SYCP1 was detected (yellow). The 6-remaning trivalents are totally synapsed. d) The single axis of an asynapsed trivalent is bound to the X chromosome single axis. At the end-to-end contact region, the SYCP1 protein was detected (arrowhead). **e–f**. γH2AX antibodies intensely labels the XY chromatin (blue), the chromatin surrounding asynapsed trivalent AEs, and is absent in the chromatin of synapsed bivalents. e) One asynapsed trivalent is associated to the XY bivalent only through chromatin where the modified histone γH2AX is detected (arrowhead). The 7-remaning trivalents are totally synapsed, not associated at all and without detection of γH2AX. f) Two asynapsed trivalents (arrows) and one asynapsed trivalent and the XY bivalent (arrowhead), are associated among them only through chromatin where the histone γH2AX is detected.

In well-preserved nuclei of heterozygote spermatocytes, each trivalent showed three points of attachment to the nuclear envelope, two of which corresponded to the distal telomeres of the metacentric chromosomes synapsed with the telocentric chromosomes and the third given by the attachment of the proximal telomeres of the telocentric chromosomes. The third point of attachment to the nuclear envelope drags the pericentromeric heterochromatin of the 3 chromosomes involved in the trivalent towards the nuclear periphery. Throughout the meiotic prophase, the distance between the three telomere attachment points over the nuclear envelope increases, so that each trivalent may have different spatial configuration options. These configurations are observed in the spermatocyte shown in Figure 5b and represented in the diagram shown in Figure 5a. In early prophase, most trivalents occupy a small space at the nuclear periphery, due to the close attachment on the nuclear envelope of the three telomeres. This configuration is modified in more advanced spermatocytes, where the distal telomeres appear attached at the opposite pole of the nuclear envelope with respect to the proximal telomere, so that the trivalent occupies almost the complete nuclear diameter. No associations between chromosomal axes were observed in any of the configurations in well-synapsed trivalents. A different situation was observed on the trivalents in which the short arms of telocentric chromosomes remain asynapsed. These associations involved an end-to-end junction between one or both single axes from two or more trivalents (Figure 5c) or between a trivalent single axis and the X chromosome single axis (Figure 5d). In both cases, as revealed by immunocytochemistry, the SYCP1 protein is detected at the end-to-end contact region (Figure 5c & d). The asynapsed trivalents also presented ectopic associations just mediated by heterocromatin from different trivalents or between a trivalent and the XY chromatin (Figure 5e & f). In these situations the heterochromatin was modified as it can be demonstrated by the presence of the histone protein γH2AX (Figure 5e & f). In 68% of mid pachytene, the single axes of partially-asynapsed trivalents established ectopic associations amongst themselves (15,7%), with the XY bivalent (27,1%) or both (23,6%) (Table 3). Only about 5% of the mid pachytene with well-synapsed trivalents presented ectopic associations, which were just mediated by heterocromatin from different trivalents or between a trivalent and the XY bivalent (Table 3). In late pachytene or diplotene spermatocytes, which mostly present just one asynapsed trivalent or the 8 trivalents completely synapsed, ectopic associations were rarely observed (not shown).

**Table 3:** **Number of partially asynapsed trivalents and the type of ectopic asociations in 2n = 32 heterocygote spermatocytes**

	Asynapsed trivalents	0	1	2	3	4	5	6	7	8	Total	%
Ectopic associations	none	35	1	3	4	3	2	0	0	0	47	33,6
	XY/triv	2	13	8	6	8	3	0	0	0	38	27,1
	triv/triv	0	0	5	7	4	4	0	0	0	22	15,7
	both	0	0	0	3	9	12	3	2	3	33	23,6
	Total nuclei	37	14	16	20	24	21	3	2	3	140	100

140 nuclear microspreadings from 2n = 32 pachytene spermatocytes were examined.

The single axes and central element of the SC were identified by immunochemistry, using, respectively, anti-SYCP3 and anti-SYCP1 antibodies (as in Figure 5c & d).

## Discussion

The nuclear architecture of spermatocytes in meiotic prophase is primarily determined by the synaptic organization of the bivalents, bound by their telomeres to the nuclear envelope and describing arc-shaped trajectories through the nuclear space. However, over this basic meiotic organization, the spermatocyte nuclear architecture is also conditioned by the individual characteristics of the chromosomes and the opportunity for interactions between their domains. The homogeneity in the morphology of the chromosomes of *Mus domesticus* 2n = 40, in addition to the numerous subspecies with reduced diploid numbers and carriers of metacentric Rb chromosomes, makes this species a convenient model to evaluate the above proposal. In this sense, the comparative analysis of the nuclear architecture of *Mus* spermatocytes with different chromosomal constitutions (2n = 40, 2n = 24 and 2n = 32) allowed us to establish which differences in nuclear organization are attributable to Rb chromosomes.

Moreover, all telocentric chromosomes of *Mus* 2n = 40 exhibit abundant pericentromeric heterochromatin that is composed of two distinct repetitive DNA sequences, the minor and major satellites. It has been shown that the major satellites with the heterochromatin protein 1alpha form clusters, creating an association of several bivalents [21]. Previous analyses have allowed us to demonstrate that associations between autosomal bivalents mediated by heterochromatin are very frequent and can be considered as being random [6]. In this work, in which two bivalents were distinguishable each time, we found that the frequency of association between them was close to 10%, coinciding with the expected rate if associations were at random. There were no significant differences in the frequency of association by spermatocyte between chromosome pairs that form Rb chromosomes (9/14 and 16/17) versus those that do not (9/16 and 14/17), nor did we find higher frequencies of association between bivalents 16 and 17 that are nucleolar and whose NOR regions are both near the pericentromeric heterochromatin. Ribosomal gene expression occurs in pachytene spermatocytes; this situation may have contributed to the association of NORs from different bivalents in the production of a common nucleolus, as has been described in somatic and meiotic cells [5, 13]. However, as *Mus* has multiple nucleolar chromosomes, we cannot rule out preferential associations among other nucleolar chromosomes [22]. In any case, frequent random associations among all bivalents would be consistent with the necessary physical proximity among their heterochromatic regions for Rb fusions to occur among any of the 19 bivalents, as has been described for the different subspecies of *Mus*[18]. On the other hand, the homology of satDNA sequences shared by the mouse telocentric chromosomes [17] might have occurred by means of multiple small exchanges among these large tracts of tandemly repeated DNA [23]. The associations among the autosomal bivalents during meiotic prophase, precisely through their heterochromatic domains, would also provide a scenario in which these frequent recombinational exchanges between non-homologous chromosomes may have occurred. A similar origin has been proposed for the concerted evolution of the ribosomal DNA spacers on non-homologous nucleolar chromosomes [24–26].

In many organisms, a meiosis-specific organization of chromosomes called the “bouquet configuration,” which is a clustering of telomeres on the inner nuclear envelope, appears to facilitate homologous recognition and alignment by concentrating all chromosomes within a limited region of the nuclear volume [27–30]. In the bouquet of the 2n = 40 spermatocytes, all of the heterochromatic ends of the telocentric chromosomes cluster together, forming a large chromocenter. Later, the progress of the prophase increases the nuclear volume and the movement of telomeres over the nuclear envelope, and consequently the great original chromocenter breaks down into the smaller chromocenters seen in the pachytene stage [6]. In contrast, during the bouquet of 2n = 24 spermatocytes, it was remarkable, that only two chromocenters were observed, a small one at the nuclear periphery and another with abundant heterochromatin toward the center of the nucleus. Therefore, two different areas for association are characteristic for these nuclei: one among the heterochromatins of telocentric bivalents, and another among the heterochromatins of metacentric bivalents. This organization continues toward the pachytene stage, where most of the spermatocytes show the telocentric bivalents tightly associated among themselves and the metacentric bivalents associated in a looser way forming two or more groups. We suggest that this new nuclear architecture would favor the progressive fusion of the remaining telocentric chromosomes. It is also possible that this narrowing of association opportunities is related to the overall trend toward production of metacentric chromosomes, as observed in the chromosomal evolution of this species [16, 31]. On the other hand, association between the heterochromatic regions of the metacentric bivalents is the scenario in which WARTS may occur. In these Rb rearrangements, described also in the karyotypes of *Mus*, two Rb metacentric chromosomes exchange their arms due to breakage and reciprocal fusion at the pericentric heterochromatin level of both [32, 33]. The described topological nuclear organization allows for the repeated encounter between chromosomal domains that may eventually experience rearrangements among them. However, under this topographic scenario it is also necessary to consider that during meiotic prophase, a programmed induction of DNA double-strand breaks (DSBs) leads to the exchange of genetic material between homologous chromosomes [34]. DSBs preferentially occurs at discrete sites called hotspots whose localization seems to be influenced by both local chromatin and higher-order chromosome structures [35]. It is not known if hotspots are preferentially localized at the heterochromatic domains of the Mus spermatocytes. However, on these meiotic prophase nuclei the full DNA repair machinery would be available, which could account for an exchange between heterologous DNA that may change the structure of the involved chromosomes.

In heterozygote 2n = 32 spermatocytes, another nuclear architecture emerges, which in this case accounts for the meeting of two different chromosome complements, one from 2n = 40 homozygotes and the other from 2n = 24 homozygotes. These hybrids, or similar, may occur naturally in any overlapping area of two subpopulations of *Mus*, one bearing the ancestral karyotype and other that has diverged by the presence of multiple Rb chromosomes. In Rb heterozygote spermatocytes, recognition and synapsis between the two parental chromosome complements occur, forming eight trivalents, three telocentric bivalents, and a sexual bivalent, similar to what has been previously described for other Rb hybrids of *Mus*[36]. Each trivalent has three points of attachment to the nuclear envelope, two corresponding to the distal telomeres and the third to the heterologous proximal ends of the telocentric chromosomes. This organization leads to the three centromeres clustering together at the nuclear periphery. Depending on the distance between the distal and proximal telomeres the trivalents may take different configurations at the nuclear space. The ultimate reason why telomeres move away or why the trivalents present different configurations remains unknown. On the other hand, in any of these configurations, the centromeric and heterochromatic domains of the very same eight pairs of telocentric chromosomes are always together in every trivalent and spermatocyte. This repeated convergence of the same pairs of heterochromatic domains should be also decisive in favoring the formation of new metacentric Rb chromosomes.

It has been reported that approximately 80% of the heterozygote spermatocytes in early prophase shows 2 to 6 trivalents with varying degrees of asynapsis between the short arms of telocentric chromosomes [20]. Chromatin surrounding these areas of asynapsis experiences changes in condensation and modifications on the underlying proteins resulting in transcriptional repression. This entire phenomenon is known as MSUC for “Meiotic Silencing of Asynapsed Chromatin” [37]. Some of the asynapsed trivalents were only connected between them or with the XY bivalent through this kind of chromatin. It has been reported that MSUC also delay the progress of meiotic prophase, which gives the opportunity that these chromosomal regions reach the synapsis what apparently it happens in many of the spermatocytes [20]. Despite of this, we observed that in 68% of mid pachytene nuclei, the single axes of partially asynapsed trivalents established ectopic associations amongst themselves (15,7%), with the XY bivalent (27,1%) or both (23,6%). Furthermore, the presence of SCP1 protein between the heterologous axes demonstrates the formation of the SC medial element and thus the possibility of recombination between heterologous chromosomal regions, which can later lead to segregational problems between those chromosomes. Therefore, the configuration of trivalents and the relationships among their unsynaptic axes impact globally the architecture of the heterozygote pachytene nuclei and possibly may have consequences for the survival of the spermatocytes. Thus, ectopic bonds could be quite deleterious to the normal progress of prophase and also to the subsequent segregation of the involved chromosomes. Spermatocytes carrying these ectopic joints may be eliminated by apoptosis in prophase or metaphase I, a phenomenon that has been observed in the Rb heterozygote male germ line [38, 39]. All of these situations could explain the reduced fertility found in Rb hybrids [38, 40–42].

Clearly, the multiple presence of Rb metacentric chromosomes in 2n = 24 spermatocytes, as in the 2n = 32 heterozygotes, determines a new organization and distribution of chromosomal domains in the meiotic prophase nucleus and consequently changes the possibilities for interchromosomal relationships as compared to those present in the 2n = 40 spermatocytes. The nuclear architecture can achieve a new optimum as have been seen in homozygote spermatocytes 2n = 24. However, in heterozygote nuclei, the areas for meeting and interaction between chromosomal domains, which actually define the nuclear architecture, are unclear and their emerging trials rather seem to produce additional instability.

On the other hand, the significant changes in the spermatocyte nuclear architecture described here allow us to better understand the difficulties that a new Rb chromosomes face in surviving meiosis, and therefore to be inherited by the progeny and propagated into the reproductive community.

## Conclusions

The Rb chromosomes pose sharp restrictions for interactions in the 2n = 24 and 2n = 32 spermatocytes, as compared to the ample possibilities for interactions between bivalents in the 2n = 40 spermatocytes.

The emergence of Rb chromosomes changes the ancestral nuclear architecture of 2n = 40 spermatocytes since they establish new types of interactions among chromosomal domains.

The associations are produced through centromeric and heterochromatic regions at the nuclear periphery among telocentric bivalents and at the nuclear center among Rb metacentric ones.

## Methods

We analyzed spermatocytes from four male three-month-old *Mus domesticus* 2n = 40 CD1 mice with all telocentric chromosomes; spermatocytes from four males of the Milano II 2n = 24 with eight pairs of homozygote Rb metacentric chromosomes; and spermatocytes from four heterozygote Rb mice 2n = 32 with eight single Rb metacentric chromosomes. The heterozygote mice were generated by mating females of the laboratory strain CD1 2n = 40 and males of the Milano II race. Male and female specimens from the original natural populations were donated to our laboratory, by Drs Carlo Redi and Silvia Garagna from the Pavia University, Italy, as part of a collaborative research proyect.

The Rb chromosomes were the following: Rb (2.12), Rb (3.4), Rb (5.15), Rb (6.7), Rb (8.11), Rb (9.14), Rb (10.13), Rb (16.17). Numbers are according the 2n = 40 standard karyotype.

Mice were maintained at 22°C with a light/dark cycle of 12/12 hours and fed ad libitum. Procedures involving the use of the mice were reviewed and approved by the Ethics Committee of the Faculty of Medicine, Universidad de Chile and by the Ethics Committee of the Chilean National Science Foundation FONDECYT-CONICYT.

### Spermatocyte squashes with 3-D preserved nuclei

Spermatocyte squashes that preserved the nuclear volume were obtained following the procedure described by Page et al. [43]. Testes were removed and fixed in 2% formaldehyde in PBS containing 0.05% Triton X-100. Pieces of tubules were placed in a drop of fixing solution on a slide. They were gently minced with tweezers, and then a coverslip was added. Exerting pressure on the coverslip squashed the cells. The slides were frozen in liquid nitrogen, and coverslips were then removed.

### Spermatocyte nuclear spreads

Spermatocyte spreads were obtained following the procedure described by Peters et al. [44]. Briefly, a testicular cell suspension in 100 mM sucrose was spread onto a slide dipped in 1% paraformaldehyde in distilled water containing 0.15% Triton X-100 then left to dry for two hours in a moist chamber. The slides were subsequently washed with 0.08% Photoflo (Kodak), air-dried, and rehydrated in PBS.

### Immunochemical identification of bivalents

The slides were incubated for 45 minutes at 37°C in a moist chamber with the primary antibodies: rabbit anti-SYCP3 1:100 (Abcam, ab15093); mouse anti-CENPA 1:200 (Abcam, ab13939); rabbit anti-H3K9me3 1:200 (Abcam ab8580); or mouse anti-phospho-histone H2AX (Ser139) 1:1000 clone JBW301 (Upstate, 05–636). Then, the slides were incubated for 30 minutes at room temperature with the secondary antibodies: FITC-conjugated goat anti-mouse IgG (1:50) (Sigma), or Texas red-conjugated goat anti rabbit IgG (1:200) (Jackson). Slides were counterstained with 1 μg/ml DAPI (4,6-diamidino-2-phenylindole). Finally, slides were rinsed in PBS and mounted in Vectashield (Vector).

### In situ hybridization

In the microspreads of the 2n = 40 spermatocytes, chromosomes 9, 14, 16, or 17 were identified by in situ hybridization using commercial probes (MetaSystems). The slides were treated for 5 minutes in 1XPBS, dehydrated in a series of 70, 80, 90, and 100% ethanol for 2 minutes each, and air-dried at room temperature. The slides with the samples and the DNA probe were denatured together at 75°C for 2 minutes. Then, the slides were incubated in a humid chamber at 41°C for 16 hours. Next, the coverslip was removed, and the slides were washed in: 0.4X SSC at 72°C for 2 minutes; 2XSSC with 0.05% Tween20 at room temperature for 30 seconds, and twice in 1× PBS for 5 minutes each. Nuclear contrast was performed with DAPI, and coverslips were mounted with Vectashield.

### Images

Observations were made using a Nikon (Tokyo, Japan) Optiphot or Olympus BX61 microscope equipped with epifluorescence optics, and the images were photographed on a DS camera control unit DS-L1 Nikon or captured with an Olympus DP70 digital camera. All images were processed using Adobe Photoshop CS5.1 software or the public domain software ImageJ (National Institutes of Health, United States; http://rsb.info.nih.gov/ij).
